# Infant Feeding in Its Relation to Health and Disease

**Published:** 1904-08

**Authors:** 


Infant Feeding in Its Relation to Health and Disease. By Louis Fischer, M.D., Visiting Physician to the Willard Parker and Riverside Hospitals, New York City. Third edition, thoroughly revised. Containing 54 illustrations, 357 octavo pages. Philadelphia: F. A. Davis Company. 1904. (Price, $2.00.)

The author presents almost a new book in his third edition, so thorough has been the revision. New chapters have been added on milk idiosyncrasies in children and on buttermilk feeding; and many changes made to afford more help to the general practitioner in the home modification of milk. The book is eminently practical and well deserves its popularity. M. J. F.

